# How pairs of insertion mutations impact protein structure: an exhaustive computational study

**DOI:** 10.1093/bioadv/vbae138

**Published:** 2024-09-27

**Authors:** Changrui Li, Yang Zheng, Filip Jagodzinski

**Affiliations:** Computer Science Department, Western Washington University, Bellingham, WA 98225, United States; Computer Science Department, Western Washington University, Bellingham, WA 98225, United States; Computer Science Department, Western Washington University, Bellingham, WA 98225, United States

## Abstract

**Summary:**

Understanding how amino acid insertion mutations affect protein structure can inform pharmaceutical efforts targeting diseases that are caused by protein mutants. *In silico* simulation of mutations complements experiments performed on physical proteins which are time and cost prohibitive. We have computationally generated the exhaustive sets of two amino acid insertion mutations for five protein structures in the Protein Data Bank. To probe and identify how pairs of insertions affect structural stability and flexibility, we tally the count of hydrogen bonds and analyze a variety of metrics of each mutant. We identify hotspots where pairs of insertions have a pronounced effect, and study how amino acid properties such as size and type, and insertion into alpha helices, affect a protein’s structure. The findings show that although there are some residues, Proline and Tryptophan specifically, which if inserted have a significant impact on the protein’s structure, there is also a great deal of variance in the effects of the exhaustive insertions both for any single protein, and across the five proteins. That suggests that computational or otherwise quantitative efforts should consider large representative sample sizes especially when training models to make predictions about the effects of insertions.

**Availability and implementation:**

The data underlying this article is available at https://multimute.cs.wwu.edu.

## 1 Introduction

We explore the impacts of double insertions on the structures of five proteins with 46, 67, 96, 99, and 125 residues by generating the exhaustive sets of all mutants with two amino acid insertion mutations. To the best of our knowledge, this and our previous preliminary work are the only efforts that computationally generate and analyze exhaustive pairs of insertion mutations. Our approach expands on our previously developed computational pipeline for generating and analyzing the exhaustive dataset of mutants with a single insertion (Turcan *et al*. 2022). We assess the impacts of insertions by studying the count of hydrogen bonds, and making use of rigidity analysis metrics for each mutant generated. This work extends significantly our previous efforts (Li and Jagodzinski 2023) by also considering the effects of insertions into alpha helices, the size and type of the inserted residues, and the locations where insertions are made that present the greatest impact to the protein’s structure as inferred via changes to count of hydrogen bonds and rigidity metrics relative to the wildtype.

We systematically compare mutants, with the aim to identify trends, and regions of double insertions which are outliers when compared to other mutants. Insights about the effects of double insertions could potentially inform therapeutic strategies for diseases linked to protein misfolding or dysfunction, and further understanding of genetic mutations that result in changes to the proteome.

## 2 Related Work

Wet-lab and computational efforts studying the effects of mutations stemming from missense mutations, and mutagenesis in general, are plentiful (Drake and Baltz 1976; Moreira *et al.* 2007; Masso and Vaisman 2008). Such work has been made possible by well-established experimental techniques such as alanine scanning including shotgun approaches which permit high-throughput analyses (Morrison and Weiss 2001) of large data sets of protein variants.

Studies to predict the effects of insertions are more sparse, but recent efforts have shown promise (Choi *et al.* 2012; Jilani *et al.* 2022; Savino *et al.* 2022). Early approaches analyzed correlations in mutational behavior between positions in multiple sequence alignments, and the results were compared with crystallographically determined contacts (Gobel *et al.* 1994). Other computational efforts include SIMPROT for simulating protein sequence evolution (Pang *et al.* 2005), and efforts which involve comparing insertions-induced structural effects with those from amino acid substitutions (Jilani *et al.* 2022). Despite these, there is still significant progress that needs to be realized, as noted by Miosge *et al.* (2015), who found major discrepancies between observed and predicted impacts made by tools such as PolyPhen2 (Adzhubei *et al.* 2013) and SIFT (Lee *et al.* 2009). In our most recent work from CSBW, we studied the effects of pairs of insertions, but did not consider the size of the inserted residues, nor the effect of inserting into *alpha*-helices, nor make use of statistical measures to discern the impact of one class of insertions over another (Li and Jagodzinski 2023).

## 3 Methods

### 3.1 Generating mutants *in silico*

We extend our previous compute pipeline from (Turcan *et al*. 2022) to generate the exhaustive set of mutants each with two insertions for a specified PDB file. We make use of an inverse kinematics inspired approach in Rosetta to first make a double insertion, followed by the use of the *remodel* and *loop* protocols to close the loops where the insertions were made (Das and Baker, 2008; Huang *et al.* 2011; Stein and Kortemme 2013). We chose to modify the existing wildtype to generate a mutant rather than predicting a mutant from sequence due to time considerations for generating the exhaustive sets of mutants with pairs of insertions. Using our approach, the mean times to generate a mutant range from 63 seconds for 1crn, to 179 seconds for 1acf (Table 1). Although state-of-the-art methods for predicting a mutant structure from sequence are available and which produce excellent mutant structure files (Baek *et al.* 2021), their run times are far greater than seconds, upwards of 30 minutes, which would make generating 3 200 400 mutants in the case of 1acf infeasible even on an HPC system.

**Table 1.** Pipeline runtime metrics (s) for generating mutants, including mean, median, standard deviation (std), minimum (min), and maximum (max) for all generated mutants with two insertions.

Runtime metric	1crn	1acf	1cdz	1csp	1hhp
Mean	63.214219	179.825711	112.742185	88.272133	81.312598
Median	56.396396	176.118459	102.119139	81.145133	54.093900
Std	33.519242	83.811859	66.628774	41.495614	71.122770
Min	6.372985	12.874839	9.140579	6.775087	9.627822
Max	608.031888	869.980323	860.965091	482.032192	843.723547

We generated datasets of exhaustive protein mutants with double insertions for the following five proteins, which were selected to represent a range of protein sequence lengths, three of which contain at least two alpha-helices to permit us to assess how insertions into alpha helices affect the protein’s structure:

- 46-residue structure of crambin, PDB 1crn
- 67-residue *Bacillus major* cold-shock protein, PDB 1csp
- 96-residue structure of the BRCT domain from a DNA-Repair protein, PDB 1cdz
- 99-residue aspartyl protease HIV-1 isolate, PDB 1hhp
- 125-residue structure of *acanthamoeba castellanni* Profilin, PDB 1acf

Thus e.g. the 99-residue PDB structure file 1hhp was elongated to 101 residues when generating each mutant. The exhaustive set of all possible protein mutants with 2 insertions for 1hhp contains $\left(\begin{array}{c} n=101\\ 2 \end{array}\right)\times 20^{2}=2\;020\;000$ unique mutant PDB structure files (Li and Jagodzinski 2023).

We made use of a 2-step approach to generate mutants. Upon a first attempt, a variety of conditions gave rise to errors which prevented our pipeline from generating a mutant with double insertions (Table 2). The errors were most often caused by a timeout of the modeling process, which was limited to 500 iterative time steps of the inverse kinematics approach in Rosetta. The reason was mostly often due to steric clashes imposed by the inserted residues, which prevented the inverse kinematic chain from closing the gaps where the insertions were being made. For those mutants that we were not able to generate during a first attempt, we invoked our pipeline again but this time increasing RAM from 16 to 24 GB. This approach allowed us to generate at least 99% of all possible mutants with two insertions for all 5 proteins (Table 2). For all computation tasks we made use of our high-throughput computing infrastructure made up of 2800 cores across 150 Dell 3660 i7-13700K GeForce RTX 3060 s, each with 12 GB of disk storage and 16–24 GB (12–22 usable) of RAM. Provisioning and scheduling of jobs was supervised by HTCondor.

**Table 2.** Mutants generated.

Protein	Mut. not generated	Total possible mut.	% Mut. not generated
1crn	2169	451 200	0.481%
1csp	326	938 400	0.035%
1hhp	19 665	2 020 000	0.974%
1cdz	77	1 901 200	0.004%
1acf	26 326	3 200 400	0.823%

Mut = mutant.

### 3.2 Metrics for characterizing mutants

In an effort to quantitatively assess the effects of double insertion mutations, we tallied the count of hydrogen bonds, and computed several rigidity-based metrics for each mutant.

#### 3.2.1 Rigidity analysis

To calculate rigidity metrics of a mutant, we made use of a C++ library for mechanical modeling and pebble game rigidity analysis of proteins (Fox *et al.* 2012). The software identifies stabilizing interactions, modeling a protein as a Body–Bar–Hinge framework, and performs a pebble game analysis on an associated graph representing the mechanical model of the Body–Bar–Hinge Framework. The output are sets of atoms that exist among rigid clusters. The use of the rigidity metrics allows us to indirectly compare the effects of pairs of insertions by comparing the rigidity profiles of the mutants, and comparing the rigidity metrics of a mutant to that of the wildtype (Andersson *et al.* 2016; Dehghanpoor *et al.* 2018).

#### 3.2.2 Hydrogen bound count

For each generated mutant we tallied the count of hydrogen bonds, which was an output of the Rosetta software. We made use of hydrogen bonds as in indirect measure of the stability of a mutant, inferring that among two mutants the one with a higher count of hydrogen bonds is the more stable one (Kollman and Allen 1972).

#### 3.2.3 Cluster configuration entropy

For each mutant we also calculated its cluster configuration entropy (CCE) score, which ranges from 0 to 1. It is a quantitative measure of flexibility using a network of stabilizing interactions and bonds in a protein, informed in part by the strength and nature of the intermolecular forces among atoms (Obukhov 1995; Radestock and Gohlke 2008; Fleck and Zagrovic 2019). Comparing two mutants, the one with a higher CCE has a greater degree of flexibility and a larger number of possible conformations it can adopt, and conversely a lower CCE configuration entropy measure refers to the mutant with a greater degree of stability.

#### 3.2.4 Rigidity order parameter

We also calculated the rigidity order parameter (ROP) of each mutant. It is a flexibility score derived from the distribution of rigid clusters of atoms that are identified when performing rigidity analysis (Radestock and Gohlke 2011; Andersson *et al.* 2016). We have made use of ROP in our past work to infer structural properties of mutants. For double insertions mutants of a protein, the one with the higher ROP is correlated to be the more stable one (Dehghanpoor *et al.* 2018; Jilani *et al.* 2022).

### 3.3 Inferring impactful pairs of insertions

We relied on a variety of count and statistical measures to identify the mutants whose double insertions were the most impactful. We relied on standard deviation measurements of our metrics, and made use of Cohen’s *d* value, to identify and distinguish mutants that were most unlike all other mutants. We assessed the effects of double insertions of inserting small versus large residues, and whether the double insertions were made into an alpha helix.

#### 3.3.1 Grouping amino acids inserted based on size

For the purpose of assessing how amino acid size affects the extent that double insertions have on protein structure, we grouped amino acids based on their volume sizes:

- Very small, VS, 60–89 Å^3^: G, A, and S
- Small, S, 108–116 Å^3^: C, D, P, N, and T
- Medium, M, 138–153 Å^3^: E, V, Q, and H
- Large, L, 162–173 Å^3^: M, I, L, K, and R
- Very Large, VL, 189–227Å^3^: F, Y, and W

These groups allowed us to define sets of pairs of insertions, from the smallest to the largest pairing including VS-VS, S-S, M-M, L-L, and VL-VL.

#### 3.3.2 Statistical measures of significance

We made use of the classical *t*-test measure (Kim 2015), as well as Cohen’s *d* value (Pommié *et al.* 2004), to ascertain quantitatively the impact of one class of insertions over another—e.g. whether the effect of double insertions involving very small residues (alanine and glycine, e.g.) is statistically different than the effect of double insertions involving very large residues (phenylalanine and tryptophan), based on a metric calculated for all mutants of a specific protein.

Cohen’s *d* measures the effect size, which indicates the standardized difference between two means:

(1) $$d=\frac{M_{1}-M_{2}}{\delta_{\text{pooled}}},$$

(2) $$\delta_{\text{pooled}}=\sqrt{\frac{\delta_{1}{\;}^{2}+\delta_{2}{\;}^{2}}{2}},$$

where in our case, *d* is the Cohen’s *d* value, *M*_1_ e.g. is the very small amino acids group for double insertions, *M*_2_ is pairwise of very large amino acids group, and $\delta_{\text{pooled}}$ is the pooled standard deviation of the two standard deviations for the corresponding groups. Small effect size of Cohen’s *d* value is 0.2, medium effect size is 0.5, and large effect size is 0.8 (Larner 2014).

## 4 Results and discussion

Our overarching goal is to locate outlier pairs of insertions (for 1crn, 1csp, 1cdz, 1hhp, and 1acf) which have a pronounced effect on the protein’s structure as inferred using the count of hydrogen bonds (HBC), CCE, and ROP. For this work, our analysis is agnostic to any functional roles of the regions where the two residues are inserted. We analyze the results based on type of inserted residues, positions, if the insertion(s) is into an *α*-helix, and size of the inserted residues.

### 4.1 Impact based on type of residue inserted

#### 4.1.1 Rigidity order parameter

**Figure 1.**
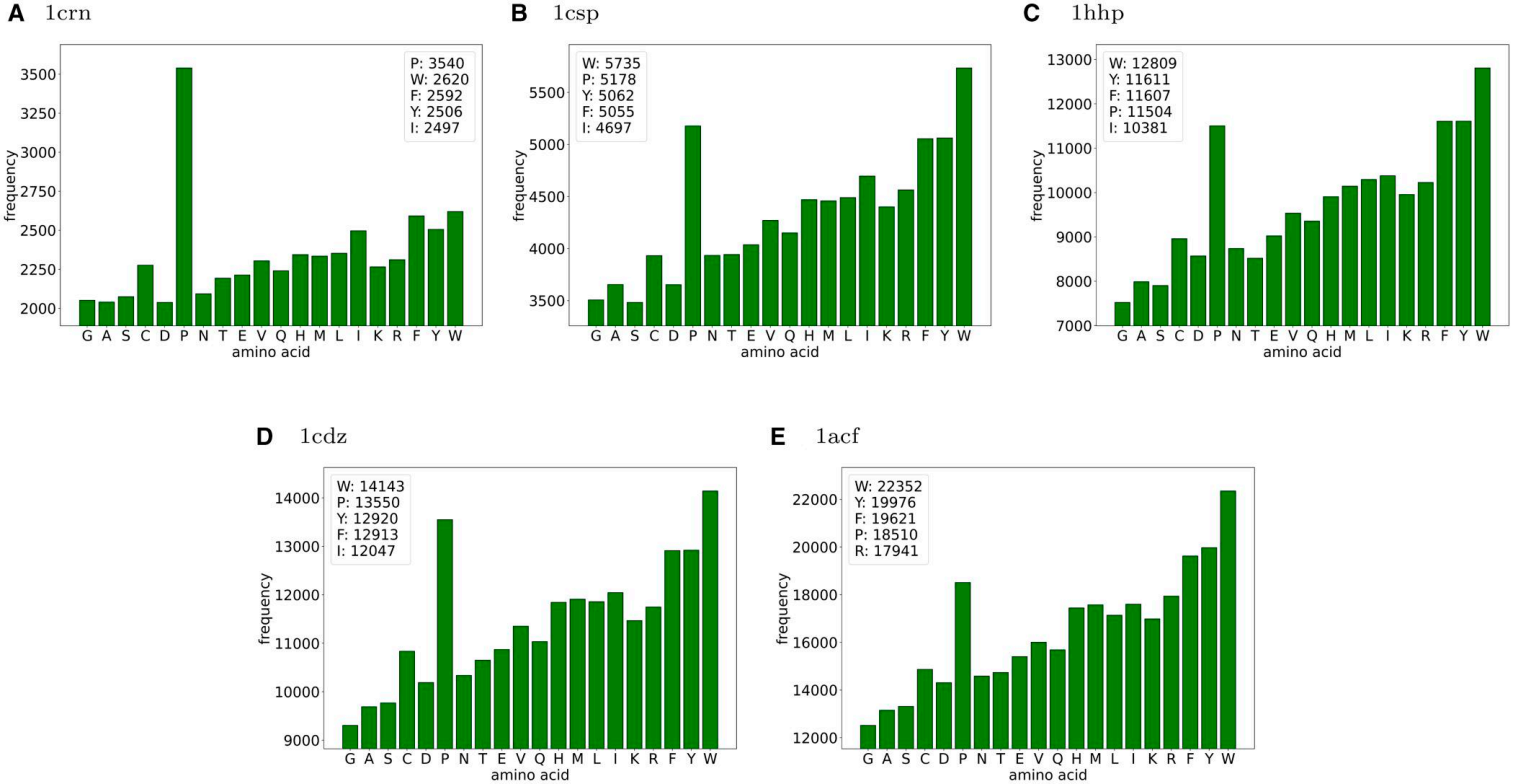
Frequencies of amino acids presenting in the sets of mutant outliers based on 2+ standard deviations for ROP for 1crn (A), 1csp (B), 1hhp (C), 1cdz (D), and 1acf (E). The legends provide the numerical counts for the five highest frequency residues.

When considering amino acids singly, we found a not unsurprising trend of smaller inserted residues generally appearing less frequently in the set of mutants that were two or more standard deviations relative to the mean in terms or ROP (Fig. 1), with Glysine (G) and Alanine (A) having the smallest frequency, and generally Tryptophan (W) and Tyrosine (Y) have the largest. Interestingly, Proline (P) is represented in the set of outliers at a much higher frequency than was expected.

#### 4.1.2 Hydrogen bond counts

**Figure 2.**
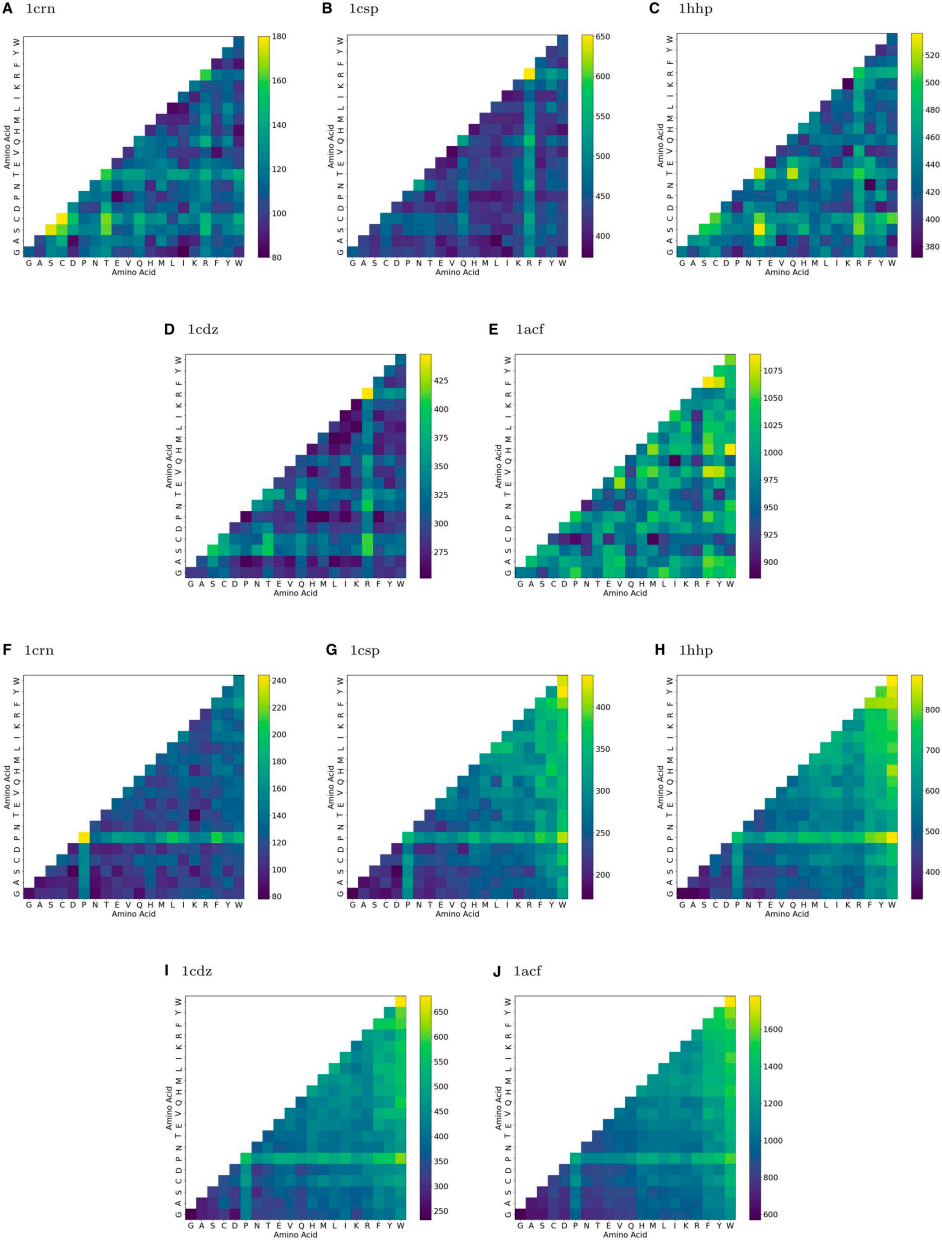
Count of pairwise amino acid insertion that appear in the set of mutants that are 2+ standard deviation outliers for hydrogen bond counts (A–F) and CCE (E–J) for 1crn, 1csp, 1cdz, 1hhp, and 1acf.

From the set of insertion mutations, the generated mutants for the five proteins presented varying counts of hydrogen bonds relative to the wildtype (Fig. 2A–E). When both inserted residues into 1crn were Cysteine (C), irrespective of insertion location, the resulting mutant was among the set of mutants more than 2 standard deviations from the average based on hydrogen bond counts. For 1csp, when both of the inserted residues were an Ariginine (R), or one was R and the other Glutamine (Q) or C, then the 1csp mutant was most often a member in the outlier set of mutants. For 1hhp, it is when one inserted residues was a Threonine (T) and the other a Serine (S) that the mutant most often was in the set of mutant outliers more than 2 standard deviations from the average count of hydrogen bonds among all mutants. For 1cdz, when both inserted residues were an Ariginine (R), the mutant was most often a member of outlier set of mutants. For 1acf, when both inserted amino acids were Phenylalanine (F) or one a Histidine (H) and the other a Tryptophan (W), the mutant most often was in the set of mutant outliers more than 2 standard deviations from the average count of hydrogen bonds among all mutants. The fact that the most impactful inserted residue was different among the five proteins is not surprising because different residues play different structural roles in different proteins, but what was surprising is that inserting R doubly or singly into 1crn, 1hhp, or 1acf had such a low impact compared to when those types of insertions were made into 1csp or 1cdz. In addition, inserting Phenylalanine doubly into 1acf had high impact compared to when those types of insertions were made into the other four proteins.

For 1hhp, we observe a relatively homogeneous distribution of frequencies for most amino acid pairings in the context of hydrogen bond count outliers. However, the pairing of Threonine (T) with S emerges as a significant outlier, distinguished by an evident high-frequency cell on the heatmap (Fig. 2C). The prominence of the TQ pairing indicates a substantial influence on the occurrence of hydrogen bonds. This frequency spike underscores the importance of the TS insertion pair and may point to specific structural configurations or interactions facilitated by Threonine residues.

For 1crn, the majority of the amino acid pairs exhibit a frequency within a moderate range. Notably, the Cysteine-Cysteine (CC) and the Serine-Serine (SS) pairs stand out with a higher frequency (Fig. 2A). This suggests that the pairing of SS and CC had a significant effect on the hydrogen bond count relative to the others pairings of insertions.

For 1csp, there is a notable high frequency of Arginine (R) in the set of mutants that are outliers, particularly evident in the RR pair context (Fig. 2B). The heatmap reveals this through a prominent light-colored cross, with intersecting vertical and horizontal bands, indicating a higher frequency of RR pairs. This pattern contrasts sharply with the surrounding darker regions and suggests a potential correlation between the prevalence of Arginine pairs and an increased count of hydrogen bonds. This observation correlates well with the fact that Arginine often engages in multiple hydrogen bonds to carbonyl oxygen (Borders Jr *et al.*1994).

For 1cdz, like for 1csp, the Arginine-Arginine (RR) residue insert pair is particularly pronounced in the heatmap of outliers (Fig. 2D).

For 1acf, the amino acid Phenylalanine-Phenylalanine (FF) pairs and Histidine-Tryptophan (HW) pairs are the most represented pairs of inserted residues among the set of mutants that are outliers (Fig. 2E).

#### 4.1.3 Cluster configuration entropy

Among all the double insertion mutants for the five proteins, we identified specific residues whose insertion produced mutants that were significantly different from all others, as inferred using CCE (Fig. 2F–J). Proline (P), when inserted along with most other residues as the second insertion, was identified to consistently have a pronounced structural impact for all five proteins. This is not surprising because Proline is known to disrupt secondary structures by inhibiting backbone orientations conducive to forming alpha helices. Next, for 1csp, 1hhp, 1cdz, and 1acf but not for 1crn, Tryptophan (W) when inserted along with most other residues as the second insertion, was also found to have a pronounced impact on the mutant’s structure relative to the wildtype. That also was not surprising, considering that Tryptophan (W) is the largest amino acid by molecular weight at 204 Daltons. Moreover, W is known to play a role in membrane protein stabilization, and especially in the context of *β*-hairpin peptides has been reported as a strong stabilizer (Santiveri and Jiménez 2010; Khemaissa *et al.* 2021).

In addition, with the increase of the size of the amino acid that is inserted, there is a positive correlation with the mutant being part of outset of outlier mutants.

### 4.2 Impact based on insertion positions

#### 4.2.1 Cluster configuration entropy

**Figure 3.**
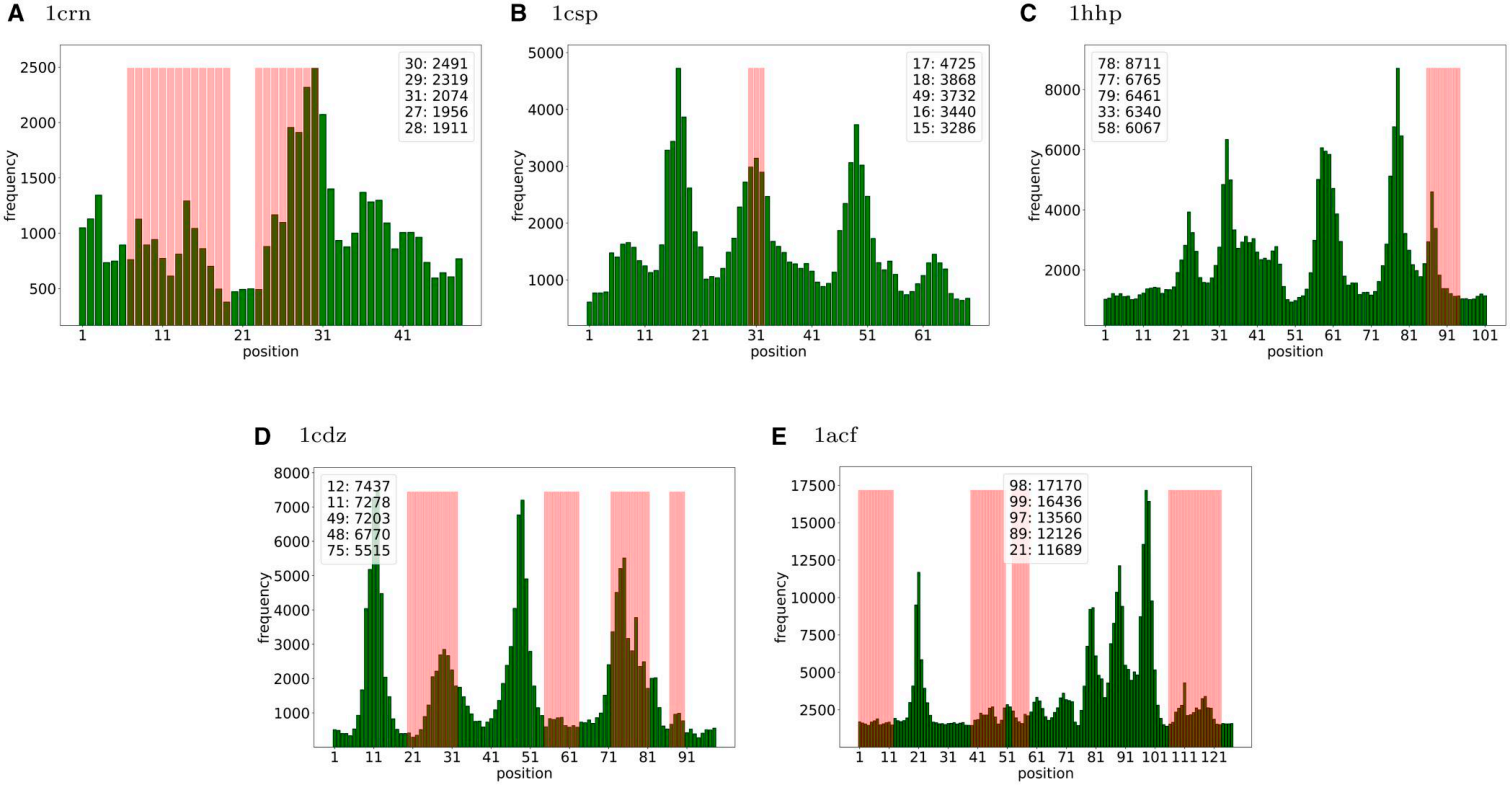
Frequencies of insertion positions that yield mutants with CCE metrics that are 2+ standard deviations from the average for 1crn (A), 1csp (B), 1hhp (C), 1cdz (D), and 1acf (E). The legends indicate the five highest frequency residues. Red indicates alpha helices.

The five proteins 1crn, 1csp, 1hhp, 1cdz, and 1acf have vastly different residue locations for insertions, which produce mutants that are outliers relative to all other mutants when using CCE as the metric (Fig. 3). That is not unexpected, considering a residue location can play a vastly different functional and structural role from one protein to another. What is unexpected is that inserting a residue into an alpha helix (shown red in Fig. 3) versus inserting into a beta sheet or non-secondary structure region appears not to correlate well with the CCE metric. For 1crn (Fig. 3A), residues 28–30 are in an alpha helix, and performing an insertion there yields 6721 mutants that are in the set of outliers according to CCE. For 1csp and 1hhp (Fig. 3B and C), however, inserting into residue locations 16–18 for 1csp, and 58 or 76–79 for 1hhp, yields mutants that are outliers according to CCE, but none of those residues are members of alpha helices. For 1cdz (Fig. 3D and E), however, inserting into residue locations 10–12, 48, and 49 in 1cdz, and into 21, 89, and 97–99 for 1acf, yielded mutants that are outliers according to CCE, but none of those residues are member of alpha helices.

### 4.3 Impact based on hydrogen bond counts for 1crn

**Figure 4.**
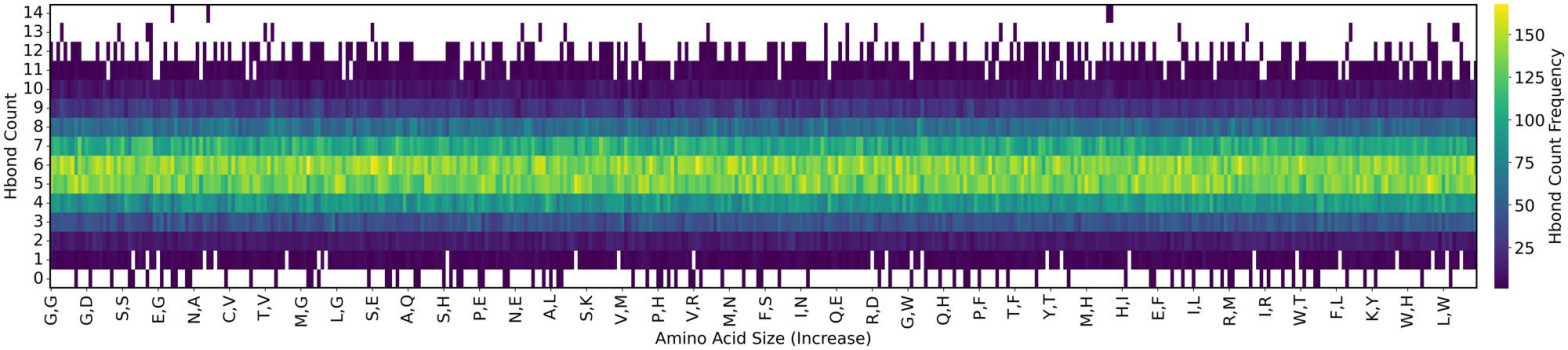
Hydrogen bound counts for each type of pairwise insertion mutation for 1crn, ordered from smallest (Glycine-Glycine GG) to largest (Tryptophan-Tryptophan WW). White specifies no such mutant.

For each mutant we tallied the count of hydrogen bonds, and sorted the insertion pairs from the smallest possible pair Glycine-Glycine (GG) to the largest Tryptophan-Tryptophan (WW) (Fig. 4). We observe that among all mutants generated, the average number of hydrogen bonds was 5.5, significantly fewer than the 9 hydrogen bonds in the wild type. However, there are a non-significant count of mutants which presented more hydrogen bonds than the wildtype, with some mutants containing as many as 14 hydrogen bonds. Alternatively, there were 26 mutants of 1crn with zero hydrogen bonds all-together, and 1298 with just one, indicating those pairs of insertions that had a significant disruption to the protein’s structure. Similar variability of hydrogen bond count across all possible pairs of insertions were observed for 1hhp and 1csp. In addition, a reasonable expectation is that inserting larger amino acids would reduce hydrogen bond formation, but the consistent distribution across the *x*-axis in Figure 4 contradicts that. It may be that inserting pairs of large amino acids has a significant-enough effect that is both local to the insertion locations as well as further away. Thus even if inserting a large amino acid does not impact the count of hydrogen bonds at or near the insertion sites, further-away regions might be still impacted to the point where hydrogen bonds are affected.

### 4.4 Impact based on insertion into an *α*-helix

To assess the extent to which an insertion into an *α*-helix impacts the protein structure, we grouped mutants by locations where insertions were made:

- *I*_XH_: mutant with one of the double insertions in an *α*-helix
- *I*_HH_: mutant with both insertions into an *α*-helix
- *I*_XX_: mutant with neither insertions into an *α*-helix

Utilizing a two-sample *t*-tests for analysis, we visualized the distribution of hydrogen bond counts for each group through normal distribution curves, illustrating both the average hydrogen bond count and its variability around the mean. We find a notable variance in the average hydrogen bond counts across different groups. Specifically, the *I*_XX_ group demonstrated the highest mean count of hydrogen bonds, followed by the *I*_XH_ group, with the *I*_HH_ group presenting the lowest mean. For proteins 1csp and 1hhp, which only include the *I*_XX_ and *I*_XH_ groups because neither of those two proteins have significant counts of alpha helices to permit creating pairs of insertions such that both are into an *α*-helix, the calculated Cohen’s *d* value suggests a minimal practical effect of inserting into *α*-helices on hydrogen bond formation. This implies that although statistically significant differences in hydrogen bond counts due to insertions within *α*-helices can be observed, the actual structural impact of these variations in 1csp and 1hhp appears to be minor. This corroborates the analysis we presented in Section 4.2.1.

**Figure 5.**
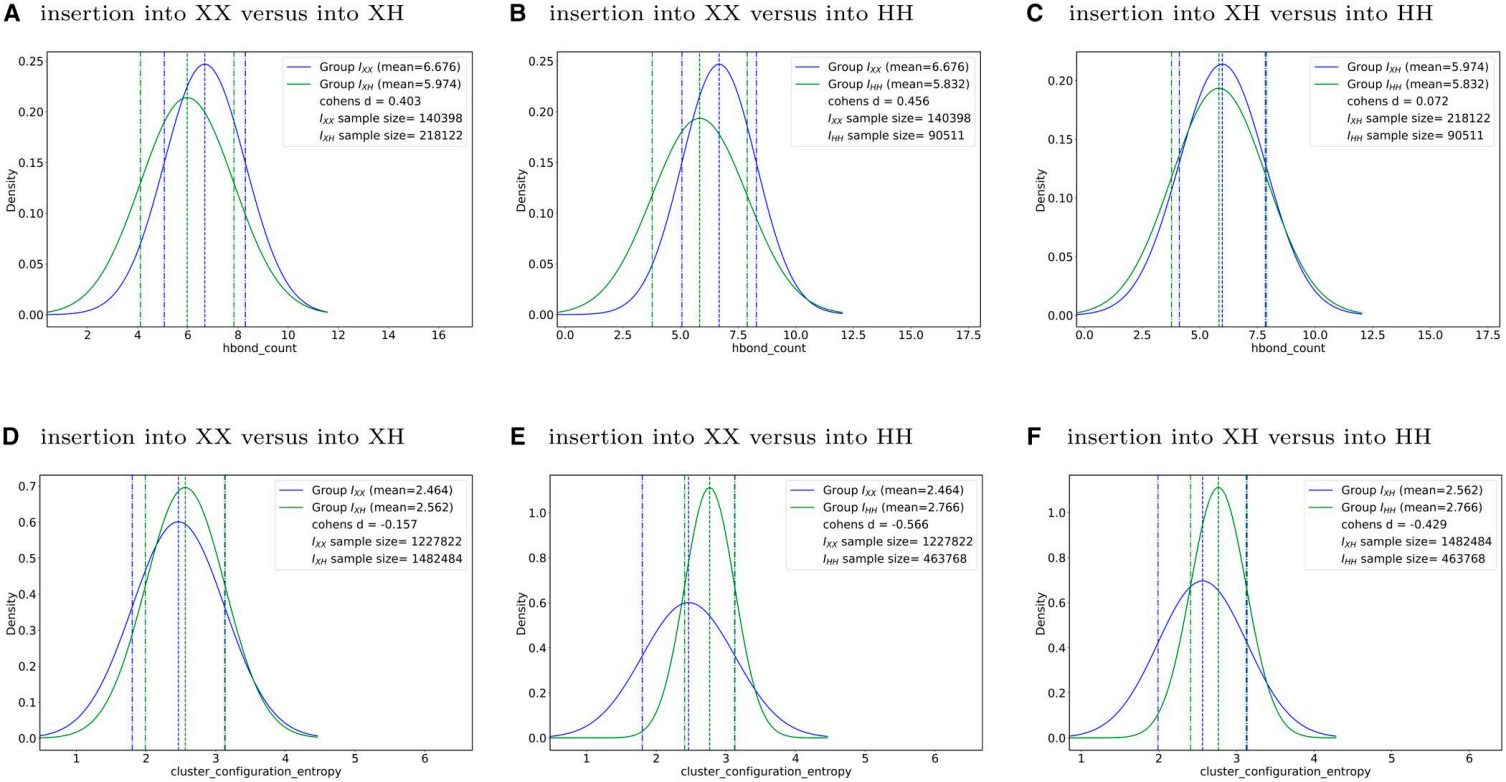
1crn Hydrogen Bond Count (A–C) and 1acf CCE (D–F), two sample *t*-test sample size and Cohen’s *d* value. Group *I*_HH_ represents both insertions are into an *α*-helix, *I*_XH_ one of the two insertions is into an *α*-helix, and *I*_XX_ neither insertion into an *α*-helix. Middle vertical dotted lines represent the mean value, the right and left vertical dotted lines represent the mean value plus and minus one standard deviation.

The situation slightly differs for 1crn, which possesses two *α*-helices of significant-enough length to yield mutants with both insertions into them. Comparing the *I*_XX_ with the *I*_XH_ group (Fig. 5A) and the *I*_XX_ with the *I*_HH_ group (Fig. 5B), we observed Cohen’s *d* values of 0.403 and 0.456, respectively. These values are higher than those noted in any other group comparison (Fig. 5C), suggesting a moderate practical effect of insertions on suppressing hydrogen bond formation. Moreover, this effect appears to increase with the number of insertions, indicating a more pronounced impact on the structure of 1crn when the insertions are made into an *α*-helix.

A similar scenario played out for 1acf, which possesses two *α*-helices of significant-enough length to yield mutants with both insertions into them. Comparing the *I*_XX_ with the *I*_HH_ group (Fig. 5E) and the *I*_XH_ with the *I*_HH_ group (Fig. 5F), we observed Cohen’s *d* values of 0.566 and 0.429, respectively. These values are also higher than those noted in comparison of XX vs XH (Fig. 5D).

### 4.5 Impact based on insertion locations and type of amino acids

**Figure 6.**
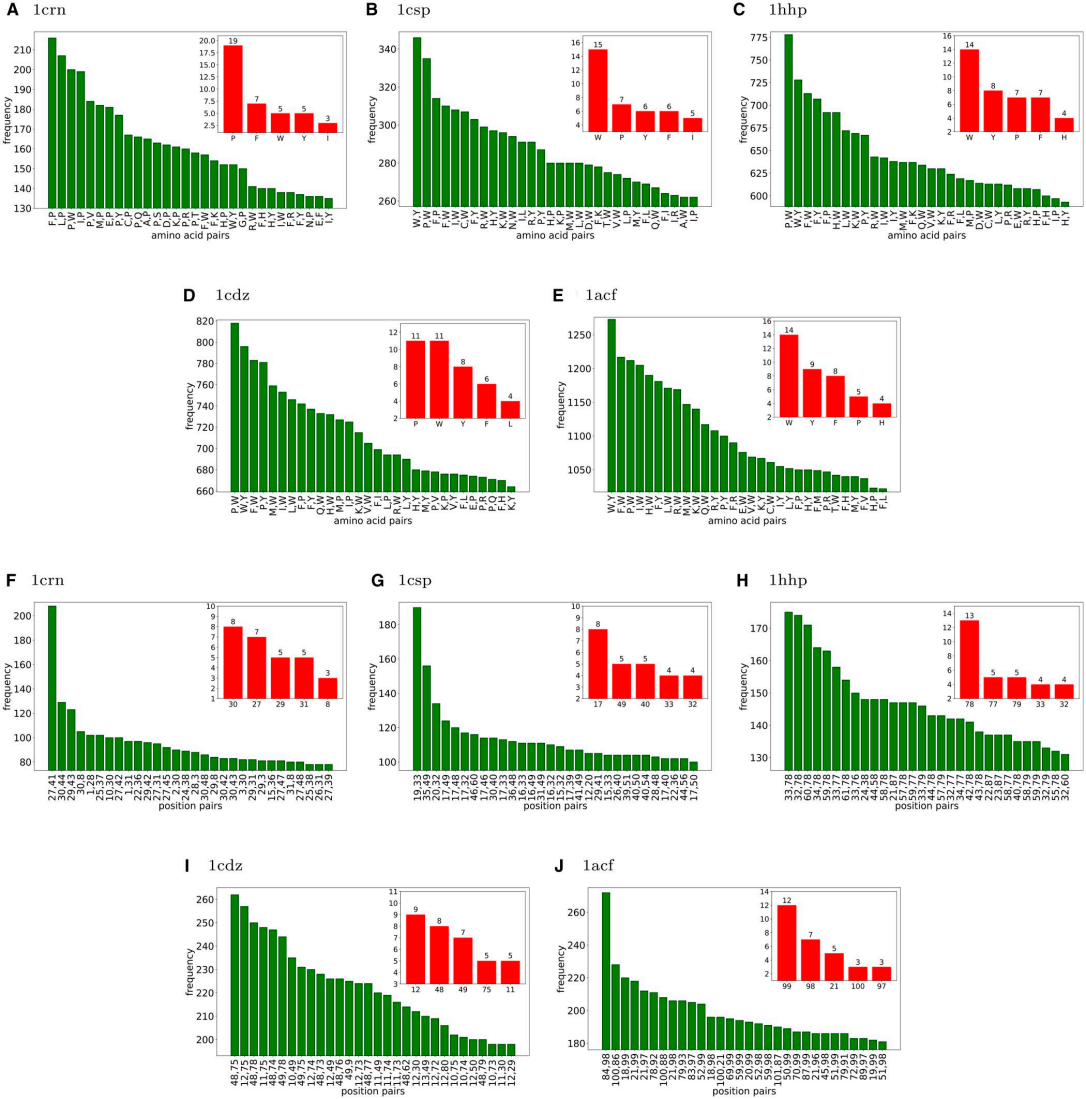
Top 30 outliers for 2+ standard deviations for ROP based on inserted pairs of amino acids (A–E), and based on insertion locations (F–J) for 1crn, 1csp, 1cdz, 1hhp, and 1acf. Subplots shows the top 5 of the inserted position or amino acid that appear among the top 30 pairs.

Irrespective of insertion location, for 1crn, it is inserting a P and a W, followed by an M and a P, and then an F as well as a P, that produce mutants that most often are in the set of outliers according to ROP (Fig. 6A). For 1csp, it is pairs of residues F and W, W and Y, and F and Y, which when inserted into the protein, produce mutants that most often are in the set of outliers relative to all other mutants of 1csp (Fig. 6B). For 1hhp, it is pairs of insertions F and W, P and W, and W and Y (Fig. 6C). For 1cdz, it is pairs of insertions P and W, P and Y, and W and Y (Fig. 6D). For 1acf, it is pairs of insertions W and Y (Fig. 6D). Across all top 30 pairs of impactful residue insertions irrespective of insertion location for the five proteins, Tryptophan (W) appears most often at 5 + 15 + 14 + 11 + 14 = 59 times, followed by Proline (P) at 19 + 7 + 7 + 11 + 5 = 49 times. But neither is Proline nor Tryptophan the most represented in the set of outliers for all five proteins.

Irrespective of which amino acids are inserted as pairs of insertions, we also tallied the pairs of insertion locations which produced mutants which are 2+ standard deviations from the average ROP score for all five proteins (Fig. 6). For protein 1crn, inserting into residue locations (27,41) yields by far the most mutants which are in the set of outliers two or more standard deviations from the average ROP score for all mutants. Among the top 30 pairs of insertion locations which are in the outlier set, residues 30, 27, and 29 appear most often (Fig. 6F). For 1csp and 1hhp, the residue pair locations into which insertions have the greatest impact on protein structure are (19,33) for 1csp, and (33,78) for 1hhp. For 1csp, residue 17 is the most represented location among the top 30 pairs of residue locations where insertions have a significant impact. For 1hhp, residues 78 is the most represented residue locations among the top 30 pairs of locations where insertions have a significant impact (Fig. 6G and H). For 1cdz, the reside pair locations into which insertions have the greatest impact on protein structure is (48,75), where residues 12, 48, and 49 are the most represented locations among the top 30 pairs of residue locations where insertions have a significant impact (Fig. 6I). For 1acf, the pair is (84,98), and the most frequent location among the top 30 pairs of locations where insertions have a significant impact is residue 99 (Fig. 6J).

### 4.6 Impact based on size of inserted amino acid pairs

To assess how the size of an inserted residue impacts a protein’s structure, and to assess similarities across ROP, CCE, and HBC as metrics for measuring the effects of mutations, we analyzed the effects of inserting very small (VS), small (S), medium (M), large (L), very large (VL), as well as VSVS, SS, MM, LL, and VLVL pairs. For those mutants which appear in the sets of 2+ standard deviations for the metrics, we normalized them against the actual counts of residues of those sizes in the proteins. We further identified the highest value for each metric among the sizes in the outlier set as well as the lowest metric value in the outlier set for all five 1hhp, 1csp, 1crn, 1cdz, and 1acf (Table 3).

**Table 3.** Comparative analysis of mutations in proteins 1crn, 1csp, 1cdz, 1hhp, and 1acf: normalization for outliers outside of two standard deviation using metrics ROP, CCE, and hydrogen bound count grouped by amino acid size which are very small (VS), small (S), medium (M), large (L), very large (VL).

	1crn			1csp			1hhp		
Group	ROP	CCE	HBC	ROP	CCE	HBC	ROP	CCE	HBC
VS	0.045754**	0.048396**	0.051335	0.037822**	0.050450**	0.092482**	0.039021**	0.048933**	0.043691
S	0.054074	0.055521	0.054386*	0.044007	0.057482	0.094138	0.046284	0.056874	0.044296*
M	0.050666	0.053756	0.049959	0.045117	0.058954	0.092819	0.047269	0.057986	0.043214
L	0.052390	0.054456	0.049610	0.048202	0.062668	0.095067*	0.050991	0.062079	0.043532
VL	0.057309*	0.059804*	0.048480**	0.056326*	0.072312*	0.093471	0.060042*	0.071644*	0.042959**
VSVS	0.039260**	0.044304**	0.053699	0.031596**	0.042586**	0.091426**	0.029493**	0.037695**	0.044607
SS	0.055391	0.056496	0.057815*	0.041409	0.054303	0.095303	0.043440	0.053686	0.044832*
MM	0.049583	0.054535	0.050473	0.044720	0.057806	0.091440	0.045889	0.056115	0.042939
LL	0.051677	0.054136	0.047507	0.051684	0.066523	0.095418*	0.053393	0.064706	0.043407
VLVL	0.064561*	0.068720*	0.044460**	0.065852*	0.083381*	0.092193	0.071792*	0.083398*	0.041888**

	1cdz			1acf		
Group	ROP	CCE	HBC	ROP	CCE	HBC
VS	0.050433**	0.037658**	0.032212	0.040944**	0.055666**	0.062186
S	0.058450	0.043538	0.033004*	0.048518	0.064401	0.061218**
M	0.059314	0.044621	0.031585	0.050825	0.067134	0.062309
L	0.062099	0.046488	0.031956	0.054968	0.072188	0.062045
VL	0.070092*	0.053118*	0.031404**	0.065056*	0.084246*	0.063867*
VSVS	0.040350**	0.030110**	0.033781	0.029740**	0.042320**	0.061498
SS	0.056094	0.041912	0.034035*	0.045236	0.060607	0.060441*
MM	0.057874	0.044119	0.031284	0.049905	0.065469	0.062665
LL	0.063549	0.047374	0.031257**	0.057607	0.075332	0.061998
VLVL	0.081052*	0.061812*	0.031303	0.078173*	0.098375*	0.066092*

* is the highest value, and **yellow the lowest value among ROP, CCE, and HBC for each group VS, S, M, L, VL and VSVS, SS, MM, LL, VLVL.

The results reveal that both ROP and CCE when used as a metric to determine impact of insertions identify the VL group of insertions, and VLVL pairs of insertions, as having the greatest impact by virtue of appearing most often in the outlier sets. This makes sense intuitively as insertions of the largest residues one might expect has the greatest impact on the structure of a protein. The HBC metric is a bit more variable. For 1hhp and 1crn, the S and SS pairs of insertions appear most among the set of outliers for HBC, but for 1csp it is L and LL. This variability we hypothesize may be explained by the fact that size does not necessarily correlate with ability to form hydrogen bonds, because inserting a small or a large residue, steric clashes aside, can affect protein structure differently depending on the structural context (insertion into the core versus high surface accessible surface area location) of the insertion, and the varying propensity of some amino acids, large or small, to engage in hydrogen bonds.

## 5 Conclusions

Our study used computer simulations to explore how pairs of insertion mutations affect protein structures. We found that the specific type and location of mutations can significantly influence the stability and function of proteins. This shows that understanding the details of mutations is crucial for predicting their impact and could help in designing treatments for diseases linked to protein misfolding.

We have pinpointed specific pairs of residue locations where insertion mutations distinctly affect the protein’s structure, as evidenced by hydrogen bond counts and rigidity metrics. These outlier residue locations where insertions have significant impact on the protein’s structure are not always contained in *α*-helices, nor is it always the same type of residue when part of an insertion pair that has the greatest impact on the protein’s structure. This suggests that approaches for inferring and predicting impactful pairs of insertion mutations need to take into account more than just secondary structures. Moreover, any machine learning models which aim to predict the effects of mutations should be trained on as large a set of mutant data as possible, owing to the variability of the effects of mutations based on the type and properties of the 20 naturally occurring amino acids, and insertion location.

## 6 Future work

We envision a variety of extensions as future work. Performing an analysis based on hydrophobic versus hydrophilic residues, might reveal that insertions involving one type of amino acid yield vastly different impacts on a protein structure than if involving another type of amino acid. Secondly, because the data set of exhaustive pairs of insertion mutations is so large, machine learning techniques might produce insights or identify trends from a high-dimensional perspective that reasons about vectors of data that include size, location, type, as well as solvent accessibility and propensity for engaging in hydrogen bonds as properties of insertions. In addition, the use of contact maps to help reason about the effects of insertions, or to help correlate how multiple features due to double insertions affect protein structure, will be explored.

## Open research

The structure files and metrics, and analysis scripts, for all generated mutants for the five proteins are available at https://multimute.cs.wwu.edu.
